# Obesity and Bone

**DOI:** 10.12688/f1000research.20875.1

**Published:** 2020-09-09

**Authors:** Joseph Proietto

**Affiliations:** 1 Department of Medicine, Austin Health, University of Melbourne, Repatriation Hospital, Heidelberg Heights 3081, Victoria, Australia

**Keywords:** Osteoporosis, Body weight regulation, Inflammation, FTO gene, Bone marrow, Osteoblast, osteoclast, osteocyte

## Abstract

Obesity and osteoporosis are both common conditions with high rates of morbidity and mortality. There is a relationship between obesity and bone. There are multiple factors that influence the risk of fracture, including the quality of bone, the risk of falls, and the padding around the bone. These multiple factors partly explain the finding that obesity protects against fractures in some sites while increasing the risk in other parts of the body. While it is well known that increased weight builds bone, there are several mechanisms related to the obese state that make the bone more fragile. These include the increased production of bone marrow fat cells at the expense of bone-forming osteoblasts, an increase in inflammatory cytokines leading to the activation of bone-resorbing osteoclasts, mutations in the *FTO* gene, and obesity-induced increased osteoblast senescence. Surprisingly, the relationship between bone and obesity is not unidirectional; there is now evidence that osteocytes are able to regulate body weight by acting as weighing machines.

## Introduction

Both obesity and osteoporosis are common conditions leading to increased morbidity and mortality. Cardiovascular disease, cancer, type 2 diabetes, obstructive sleep apnoea, osteoarthritis, psychosocial problems, hypertension, fatty liver, and infertility are also all caused by or aggravated by obesity. Of these, it has been shown that cardiovascular disease and cancer account for the highest mortality ^1^. Osteoporosis increases the risk of fractures and results in increased mortality ^2^ and morbidity, including increased pain and reduced mobility, giving a reduction in quality of life ^3^. The very first link between obesity and bone was the common belief that an individual with obesity was heavier because he was “big boned” ^4^. Although this is a common myth, science has revealed a surprising bidirectional relationship between excess fat and bone. Furthermore, Ilich and colleagues have proposed the existence of osteosarcopenic obesity linking obesity and osteoporosis to muscle weakness ^5^. In their formulation, they discuss possible mechanisms, such as mesenchymal cell development, linking these three conditions. Osteosarcopenic obesity occurs in old age, but it has been shown that even preschool obesity is associated with an increase in fracture risk in teenage children ^6^. This review will briefly discuss how excess fat damages bone and how bone contributes to the regulation of body weight.

## How does obesity influence bone?

Weight-bearing exercise increases bone density ^7^, so pressure from excessive weight may strengthen bone ^8^. Consistent with this, significant weight loss achieved with bariatric surgery results in bone loss ^9^, which can be mitigated by exercise ^10^. However, despite more bone mass, obesity confers an increased risk of osteoporosis ^11^ and bone fractures ^12^, at least in some sites. Compston and colleagues ^13^ followed 52,939 post-menopausal women for 3 years. During that time, 3,628 reported a fracture. BMI was protective for hip with a hazard ratio (HR) of 0.8 (95% confidence interval [CI] 0.71–0.90), spine with a HR of 0.83 (95% CI 0.76–0.92), and for wrist with a HR of 0.88 (95% CI 0.83–0.94) (HR with 95% CI for each increase in BMI of 5 kg/m ^2^). However, excess weight increased the risk of ankle fractures (HR 1.05 [1.02–1.07]) ( *P* <0.01). For pelvic and rib fractures, they found a non-linear relationship with inverse associations at low BMI/body weight ( *P* = 0.05) and positive associations at high values ( *P* = 0.03). Interestingly, Liu and colleagues in a cross-sectional study involving 471 women divided into normal, overweight, and obese groups found that there is a threshold effect of fat on bone density. They concluded that while body fat below 33% has a positive relationship with bone density, for body fat above 33% the relationship between fat mass and bone is negative for most skeletal sites ^14^. So extra weight builds bone and extra fat padding protects the bone during a fall. So why is there an increased rate of fracture in some sites? It appears that obesity causes bone to be of poor quality.

## How can obesity damage bone?

Several mechanisms have been proposed for the deleterious effects of obesity on bone:

1. Replacement of osteoblasts by fat cells in bone marrow
2. Increased inflammation present in obesity
3. Mutations in the fat mass and obesity-associated ( *FTO*) gene leading to weight gain and bone fragility
4. Increased metabolism and accelerated senescence in stromal stem cells

### Replacement of osteoblasts by fat cells in bone marrow

**Figure 1.**
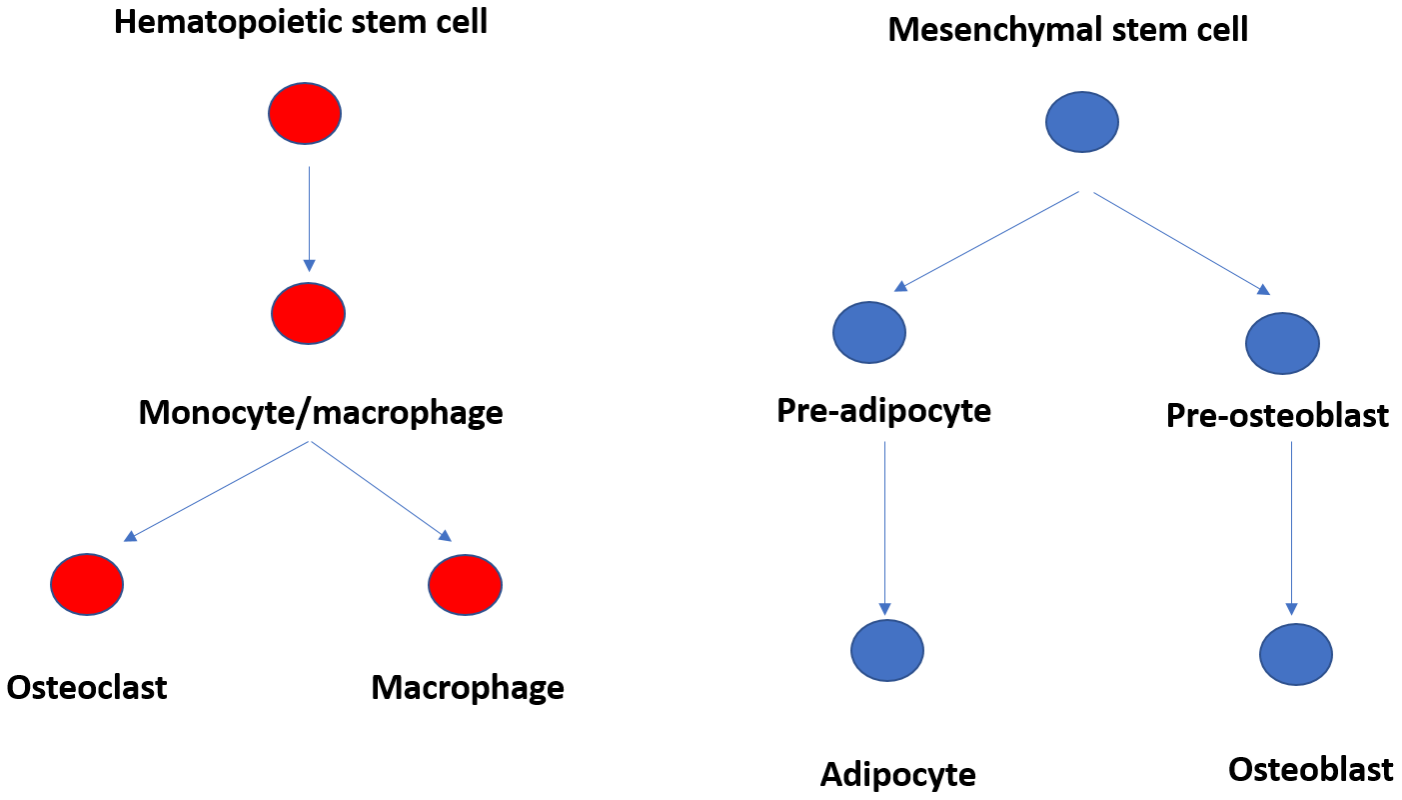
Ontogeny of fat, osteoblast, and osteoclast cells in bone marrow.

Clinically, it has been found that situations leading to bone loss such as old age, thiazolidinedione treatment, and prolonged corticosterone treatment are associated with increased fat in bone marrow ^15^. Adipocytes and osteoblasts are both derived from the same bone marrow-derived mesenchymal cell precursor ( Figure 1). Increasing adipocyte differentiation decreases osteoblast differentiation and vice versa ^16, 17^. The signals that cause the stem cells to differentiate into one or the other include the transcriptional activator PPARγ, which can move the differentiation towards the adipocyte lineage ^18^. Other triggers include leptin ^19^ and the Wingless-related Integration site (Wnt) signalling glycoproteins. Wnt signalling is an ancient controller of cell fate determination, cell migration, and more during embryogenesis ^20^. It has been found that Wnt activation stimulates osteoblast formation and inhibits adipocyte differentiation. Secreted frizzled-related protein 1 (sFRP-1), an inhibitor of Wnt signalling, is increased in adipocyte stem cells ^21^. sFRP-1 has been shown to be increased in mild obesity, leading to increased adipocyte formation, but interestingly is reduced in severe obesity, possibly to limit further adipocyte formation ^22^.

### Increased inflammation present in obesity

Obesity is now known to be a state of low-grade inflammation associated with elevated levels of cytokines ^23, 24^. Cytokines have been shown to increase bone resorption. They do this primarily by enhancing osteoclast activity. Monocyte chemotactic protein-1 (MCP-1), tumour necrosis factor (TNF)-related apoptosis-inducing ligand (TRAIL), receptor activator on nuclear factor kB ligand (RANKL), interleukin 6 (IL-6), TNFα, and TNF super family 14 (TNFSF14)/LIGHT activate genes involved in the formation of bone-resorbing osteoclasts, as reviewed by Faienza and colleagues ^25^.

### Mutations in the FTO gene leading to weight gain and bone fragility

The *FTO* gene has been linked to overweight and obesity ^26^. FTO is an RNA demethylase and has also been linked to increased risk of osteoporosis ^27^. Deletion of *FTO* in mice using the CRE/LOX system in osteoblasts leads to increased death of osteoblasts and bone loss. It has been shown that FTO functions to increase the stability of mRNA-encoding proteins that protect osteoblasts from genotoxic damage ^28^. Thus, those individuals whose obesity is caused by a mutation in the *FTO* gene are at increased risk of osteoporosis because of increased susceptibility of osteoblasts to cell death.

### Increased metabolism and accelerated senescence in stromal stem cells

The next possible mechanism for obesity-related bone fragility is proposed obesity-induced hypermetabolism leading to senescence in osteoblasts. Tencerova and colleagues ^29^ studied 54 healthy men whom they divided into lean (BMI 22.9 ± 0.3 kg/m ^2^), overweight (BMI 28.0 ± 0.4 kg/m ^2^), and obese (BMI 36.1 ± 0.8 kg/m ^2^). Using DXA scanning, they showed that the men with obesity had an increase in total hip bone mineral density but no difference in lumbar spine compared with lean men. Subjects with obesity also had a reduction in bone turnover. Because the presence of inflammation leads to increased bone turnover, the authors proposed that this means that the bone marrow is protected from the generalised increase in inflammation seen in obesity. They obtained bone marrow-derived mesenchymal stem cells (BM-MSCs) from these men and performed RNA sequencing. They found that 129 genes were upregulated and 168 genes were downregulated in men with obesity compared to lean men. Gene ontology analysis showed that the genes that were upregulated in obesity are genes involved in cell differentiation while those that were downregulated were genes for glucose metabolism and response to hypoxia. Further analysis of glucose metabolism genes showed an increase in oxidative phosphorylation while genes for anaerobic glycolysis were downregulated. Interestingly, they also found upregulation of PPARγ and other markers of differentiation into fat cells in obese compared with lean BM-MSC cultured cells. Obesity is known to be associated with insulin resistance in peripheral fat cells, muscle, and liver. In contrast, insulin resistance was not found in BM-MSCs from subjects with obesity. The BM-MSCs were then divided into those expressing the insulin and leptin receptors and those not expressing them. They showed that those expressing insulin and leptin receptors were more likely to differentiate into adipocytes while those that were negative for these receptors were more likely to differentiate into osteoblasts. It had previously been shown that osteoblast-like cells prefer glycolysis for ATP production while adipocyte-like cells prefer mitochondrial oxidative phosphorylation ^30^. High levels of oxidative phosphorylation can increase the production of toxic reactive oxygen species (ROS), which may drive senescence ^31^. When they compared insulin receptor- and leptin receptor-positive BM-MSCs from their patients, they found that these cells had an increase in the expression of senescence-associated genes when compared to cells that were negative for these receptors. Cultures of BM-MSCs from men with obesity contained a higher number of senescent cells and increased ROS production. It was concluded that enhanced insulin signalling and enrichment of adipocyte progenitor cells in the marrow of individuals with obesity lead to increased oxidative phosphorylation and increase ROS production, leading to a senescent phenotype, which results in poor bone formation.

## How does bone influence body weight?

It appears that bone can assess body weight. Jansson and colleagues ^32^ placed fluid-filled capsules into the abdomens of Sprague-Dawley rats and C57BL6 mice with diet-induced obesity to equal approximately 15% of their body weight. Control rodents had empty capsules inserted into their abdominal cavities (3% of their body weight). Increased loading suppressed body weight in both rats and mice with the difference in weight compared to control rodents already present from day 2 post-surgery. After 14 days of loading, 80% of the increased load was counteracted by reduced body weight. At the end of the experiment, body weight plus capsule weight was similar in the load and control rodents.

MRI imaging on day 14 showed that increased loading reduced the amount of white adipose tissue with no difference in brown adipose tissue. Not surprisingly, leptin levels were lower, consistent with the reduced adipose tissue mass. Weight loss was due to reduced food intake in both rats and mice. There were no changes in VO _2_ max, respiratory quotient, or muscle activity, suggesting that all of the effects of capsule loading were due to a reduction in energy intake. This was further confirmed by the fact that pair fed rodents decreased their body weight to the same extent as load rodents.

The effects of loading to suppress weight were shown to be independent of leptin when they showed that leptin-deficient ob/ob mice respond similarly to wild-type mice to capsule load. In any case, the reduction of leptin should have led to an increase in energy intake, not in the observed reduction in intake, so what is the signal?

It was known that osteocytes can sense dynamic short-term high-impact bone loading, so the authors hypothesised that osteocytes may also sense chronic static bone loading due to increased body weight. To test this hypothesis, mice deficient in osteocytes were loaded with the capsules. It was found that the control mice lost weight as shown before but the equally loaded osteocyte-deficient mice did not. So, load sensing is mitigated by the osteocyte.

The following question then arose: how does the osteocyte send the signal of increased load to the hypothalamus? In previous studies, dynamic short-term high-impact loading was shown to reduce the expression of *Sost*, the gene coding for sclerostin. However, there was no difference in sclerostin levels. There was also no change in carboxylated and undercarboxylated forms of osteocalcin, fibroblast growth factor 23, and lipocalin 2. In addition, deficiency of ghrelin, glucagon-like peptide 1, the melanocortin 4 receptor, or the oestrogen receptor did not remove the effect. This leaves the possibility of a neuronal signal from osteocytes and the hypothalamus. To begin to investigate this, the authors measured urinary noradrenaline/creatinine ratio and serum choline level but found no differences. It was concluded that the osteocyte can sense chronic weight load and can send an unknown signal that can fairly accurately correct body weight.

## Conclusion

There is a bidirectional complex relationship between obesity and bone. The advances in understanding how obesity leads to increased fragility in bone may lead to new treatments to improve bone strength. Finally, finding the signal that osteocytes send to the hypothalamus may lead to new approaches in the treatment of obesity.
